# Synthesis of New 1,3,4-Thiadiazole and 1,2,3,4-Oxathiadiazole Derivatives from Carbohydrate Precursors and Study of Their Effect on Tyrosinase Enzyme

**DOI:** 10.3390/molecules17078378

**Published:** 2012-07-11

**Authors:** Mohamed M. El-Sadek, Seham Y. Hassan, Huda E. Abdelwahab, Galila A. Yacout

**Affiliations:** 1 Chemistry Department, Faculty of Science, Alexandria University, Alexandria 21231, Egypt; Email: sehamyassen@yahoo.com (S.Y.H.); huda_eid@yahoo.com (H.E.A.); 2 Biochemistry Department, Faculty of Science, Alexandria University, Alexandria 21231, Egypt; Email: galila_69@yahoo.com

**Keywords:** carbohydrazide, thiosemicarbazone, thiadiazole, oxathiadiazole, tyrosinase

## Abstract

5-(1,2,3,4-Tetrahydroxybutyl)-2-methylfuran-3-carbohydrazide (**2**) was condensed with a variety of ketones to afford carbohydrazide derivatives **3**–**6**. Acetylation of **3**–**5** afforded the acetyl derivatives **7**–**9**, while periodate oxidation of **3**–**6** afforded the formyl derivatives **10**–**13**. Acid catalyzed condensation of thiosemicarbazide or *o*-tolylthiosemicarbazide with the prepared aldehydes **10**–**12** gave thiosemicarbazone derivatives **14**–**19**. Cyclization of the latter with acetic anhydride afforded 4,5-dihydro-1,3,4-thiadiazolyl derivatives **20**–**25**. On the other hand, condensation of *p*-tosylhydrazine with the prepared aldehydes **10**–**12** afforded *p*-tosylhydrazone derivatives **26**–**28**. Cyclization of **26**–**28** with acetic anhydride afforded 1,2,3,4-oxathiadiazole derivatives **29**–**31** respectively. Moreover, the obtained results regarding to the effect of some of the prepared compounds on tyrosinase enzyme showed that the majority of these compounds having an inhibitory effect; especially compounds **12**, **16**, **17**, and **28**.

## 1. Introduction

It was shown that substituted 1,3,4-thiadiazoles exhibit antimicrobial [1] and antitubercular [2,3,4] activities, while other compounds act on the CNS as anticonvulsants [5,6,7] or as antidepressant and anxiolitic [8] agents. A family of selective 1,3,4-thiadiazole phosphodiesterase inhibitors [9], and selective orally active cyclooxygenase-2 inhibitors [10] were reported. Moreover, many reports indicate that acylthiosemicarbazides and their corresponding cyclized 1,3,4-thiadiazole derivatives possess anti-inflammatory [11,12,13] and analgesic [14] activities. 1,3,4-Thiadiazoles are thus a group of heterocycles whose derivatives are important in industry, medicine and agriculture [13,15,16,17,18,19,20,21]. Accordingly, in continuation of our work in this area [22,23,24,25,26,27], a variety of heterocyclic derivatives have been prepared from saccharide derivatives, involving some new thiadiazoles, oxathiadiazoles, and their chemistry and effect of the derivatives on the enzyme tyrosinase was studied [28,29,30,31], which is the rate limiting step in melanin biosynthesis [32]. In humans, the main role of the melanins is photoprotection of the skin by absorbing UV radiation that causes DNA damage and the formation of reactive oxygen species (ROS). Human deficiency in melanin causes serious disorders like oculocutaneous albinism and vitiligo. There has also been great interest in the involvement of melanins in malignant melanosomes, the carcinogenic tumors of the skin. Melanoma is most commonly found on the skin, but around 10% arise in the eyes [33]*.*

In addition, tyrosinase is involved in dopamine biosynthesis, which has been shown to be involved in the control of movements, and the signaling of errors in the prediction of reward, motivation, and cognition. Cerebral dopamine depletion is the hallmark of Parkinson’s disease [31]. Other pathological states have also been associated with dopamine dysfunction, such as schizophrenia, autism, and attention deficit hyperactivity disorder [32].

## 2. Results and Discussion

### 2.1. Chemistry

**Scheme 1:**
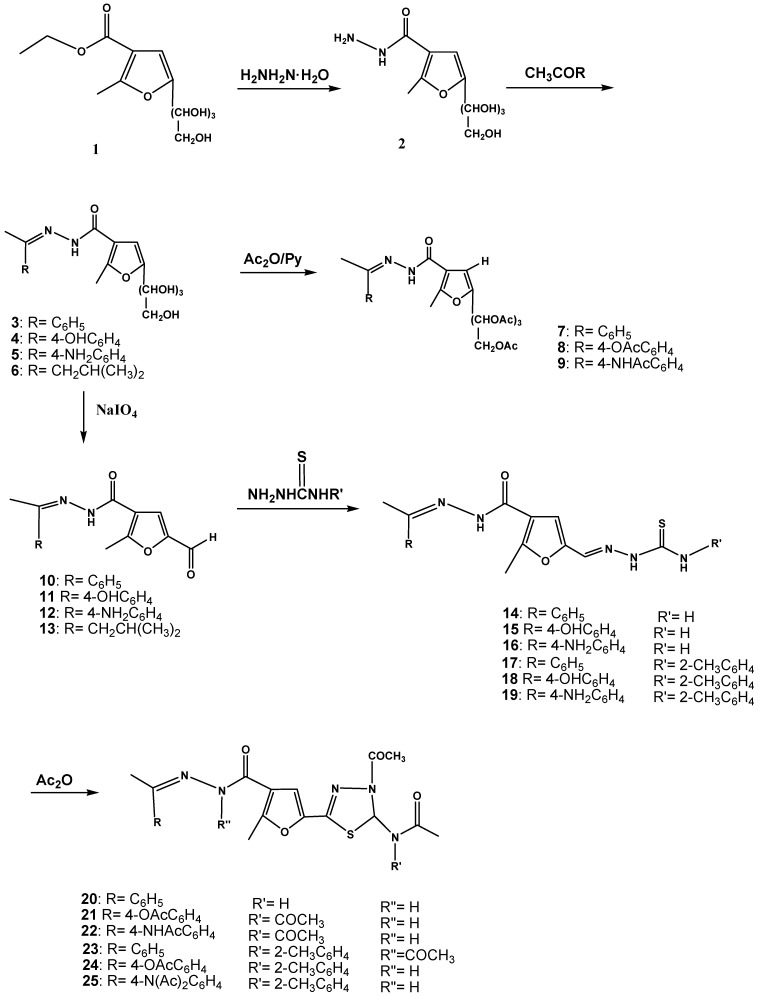
Synthesis of 1,3,4-thiadiazole derivatives.

Ethyl 5-(1,2,3,4-tetrahydroxybutyl)-2-methylfuran-3-carboxylate (**1**) [34] was prepared, then boiled with hydrazine hydrate to give carbohydrazide **2** [35], which when condensed with a variety of ketones afforded carbohydrazides **3**–**6** in 64–96% yield (Scheme 1).

The structure of hydrazones **3**–**6** was proven by their ^1^H-NMR spectra, which showed the NH proton as a singlet at δ 9.87–9.20, the proton at position-4 in the furan ring as a singlet at 7.27–6.57 ppm, the 1'-OH proton at 5.06–5.05 and the rest of the sugar protons at the 4.58–4.28 range. The methyl protons at position-2 in the furan ring appeared as a singlet at δ 2.44–2.21 ppm; additionally the disappearance of the two NH_2 _protons was observed (see Experimental). It was observed that the C-1' hydroxyl proton of compounds **3**–**6**, resonates at lower field (5.06–5.05 ppm) than the rest of the sugar protons. Electronic deshielding by the adjacent base residue undoubtedly is a major factor in causing these signals to appear at low field. Additional deshielding might also arise through the formation of an intramolecular hydrogen bond with the oxygen of the furan ring. Hydrogen bonding of this type was suggested in polyhydroxyalkyltriazole analogs [36] and polyhydroxyalkylpyrazolo-[3,4-b]-quinoxalines [37] having the D-arabino configuration of the side chain. In addition, the mass spectra of compounds **5** and **6**, as examples of the series, showed the corresponding molecular ion peaks at *m/z* 377 and 342, respectively. On the other hand, acetylation of **3**–**5** afforded the corresponding acetyl derivatives **7**–**9** in 45–86% yield (Scheme 1).

The ^1^H-NMR spectra of compound **8** and **9** showed the disappearance of the OH protons in the sugar region, the *O*-acetyl protons at δ 2.43 and 2.23 ppm, respectively, and peaks at 2.59, 2.03 for the *N*- acetyl protons, respectively (for the other protons see the Experimental). Periodate oxidation of compounds **3**–**6** afforded the corresponding formyl derivatives **10**–**13**, in 36–65% yields (Scheme 1).

^1^H-NMR spectra of compounds **10**–**12** showed the NH proton as a singlet at δ 9.32, 8.91 and 9.36 ppm. respectively, and the formyl group proton as a singlet at δ 9.87, 9.34, and 9.44 ppm, respectively (for other protons see the Experimental). The mass spectrum of compound **13**, showed the molecular ion peak at *m/z* 250, which was also the base peak. 

Acid catalyzed condensation of thiosemicarbazide or *o*-tolylthiosemicarbazide with the prepared formyl derivatives **10**–**12** gave thiosemicarbazone derivatives **14**–**19** in 43–99% yield (Scheme 1). The mass spectra of compounds **14**, **16**–**18** showed the molecular ion peaks at *m/z* 343, 358, 433 and 449 respectively. Cyclization of the prepared compounds **14**–**19** with acetic anhydride afforded 1,3,4-thiadiazole derivatives **20**–**25** in 40–73% yield (Scheme 1).

**Figure 1:**
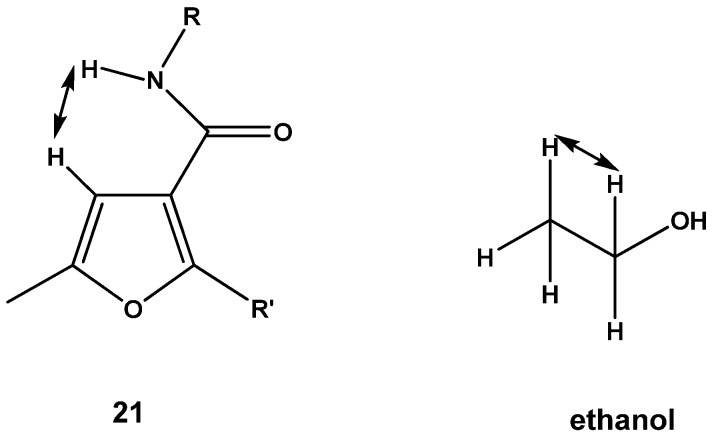
The distance between NH proton and H-furan is equal to the distance between the methylene protons and the methyl protons in the ethanol molecule.

**Figure 2:**
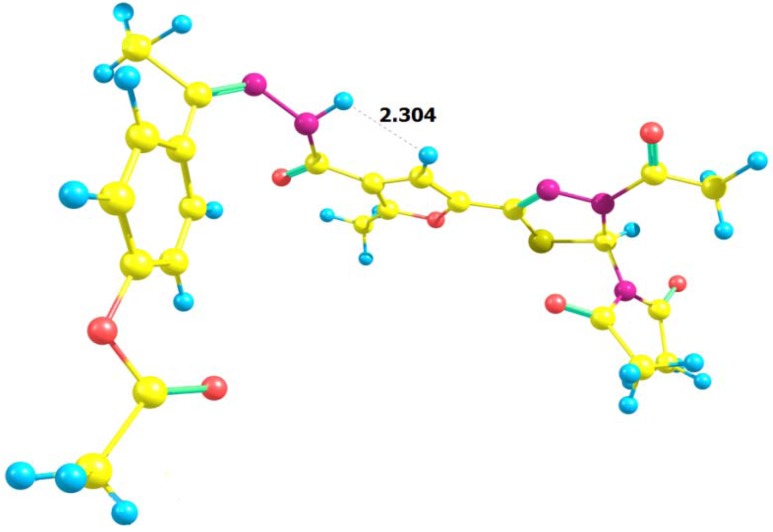
Orca Computational Chemistry program of the compound **21**.

The ^1^H-NMR spectra of compounds **20**–**22** showed the disappearance of the NH_2_ protons and CH=N proton. Instead, the N-Ac methyl protons appeared as a singlet. Interestingly, it was noted that the ^1^H-NMR spectra of compounds **21** and **22** showed the proton at position-4 in the furan ring as a doublet signal at δ 6.69 and 7.22 instead of a singlet signal due to the long rang interaction between H-furan and the NH proton of the amide group. However a theoretical study of the NMR of compound **21** was attempted whereby the stable conformer of this compound was first established using the universal force field UFF molecular mechanics method (Table 1). After that the {B3LYP/6-31G (d)} density functional approach was used to fine tune the geometry of the compound. The Orca computational chemistry program was used in this step. According to the calculation the distance between the NH proton and the H-furan is equal to 2.304 Å, which is the same value of the distance between the methylene protons and the methyl protons in the ethanol molecule. In the same way, H-furan appeared as a doublet due to the coupling interaction with the NH proton, while the proton of the NH group appears as a singlet, so the question is why the interaction with the H-furan didn't affect the signal of (NH) proton. This is attributed to the ionization factor [38] (Figure 1 and Figure 2). 

**Table 1:** The proton NMR isotropic shift of compound **21** calculated theoretically at the level 6-311G (d, p) using the Orca program and compared with the experimental values.

Proton	Calculated	Experimental
**H-1**	2.42	2.24
**H-2**	2.62	2.27
**H-3**	7.02	8.22
**H-4**	6.62	6.69
**H-5**	7.82	7.06
**H-6**	2.22	2.14
**H-7**	2.39	2.34
**H-8**	7.32	7.23–7.26
**H-9**	7.32	7.23–7.26
**H-10**	2.22	2.21
**H-11**	2.39	2.34

**Scheme 2:**
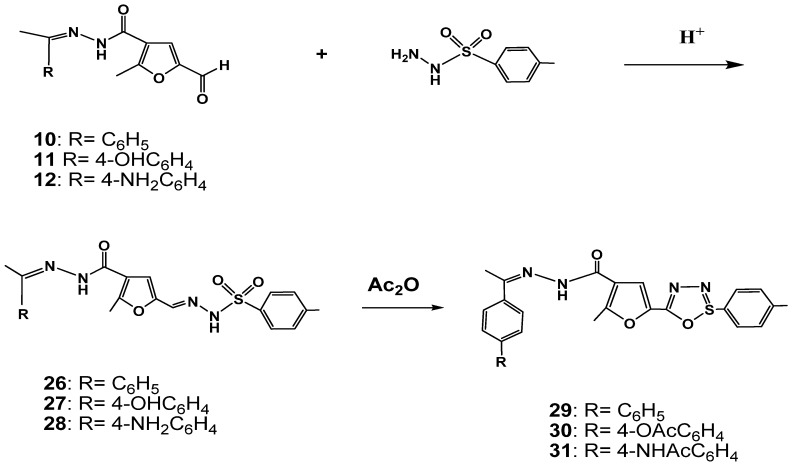
Synthesis of oxathiadiazole derivatives.

The mass spectra of compounds **20** and **22** showed the molecular ion peaks at *m/z* 427 and 526, respectively. The mass spectrum of compound **23** showed the molecular ion peak at *m/z* 560. In addition, condensation of *p*-tosylhydrazine with the formyl derivatives **10**–**12** afforded *p*-tosylhydrazone derivatives **26**–**28** respectively in 42–83% yield (Scheme 2). The ^1^H-NMR spectra of compounds **26** and **27** showed the disappearance of the aldehyde proton. The two NH protons showed as a singlet at δ 9.36, 9.71, and 9.36, 10.47, respectively, the CH_3_ protons of the *p*-tolyl moiety as a singlet at δ 2.36 and 2.32 ppm, respectively (see Experimental part). The mass spectra of compounds **26** and **27** showed the molecular ion peaks at *m/z* 438 and 454, respectively.

Similarly, cyclization of these hydrazones **26**–**28**, with acetic anhydride afforded 1,2,3,4-oxathiadiazole derivatives **29**–**31** in 32–54% yield (Scheme 2). The ^1^H-NMR spectra of the compounds **29** and **31** showed the disappearance of both the CH=N and the NHSO_2_ proton signals. The ^1^H-NMR spectra showed the CH_3_ protons of the *p*-tolyl group as a singlet at δ 2.40, 2.49 ppm, the CH_3_-C=N protons as a singlet at δ 2.05, 2.09 and the CH_3_-furan protons as a singlet at δ 2.40, 2.49, respectively. The mass spectra of compounds **29** and **30** showed the molecular ion peaks at *m/z* 420 and 478, respectively.

### 2.2. Biological Activity Assay

Tyrosinase was prepared from mushrooms in a phosphate buffer (50 mM, pH 6.0) according to the method of Yang and Robb [39], and the obtained supernatant after centrifugation was used as a source of enzyme. 

#### 2.2.1. Enzyme Activity Assay

The activity of the prepared enzyme solution was determined by following the formation of dopachrome spectrophotometrically at 30 °C, after addition of 50 μL enzyme preparation to a cuvette containing 1.2 mL phosphate buffer (50 mM, pH 6.0) and 0.8 mL L-Dopa (10 mM), the solution was immediately mixed and the increase in absorbance at 475 nm (indicating the formation of dopachrome) was recorded using UV-20100-spectrophotometer. Blank experiment was carried out as mentioned above using 50 μL of buffer instead of enzyme preparation [40].

#### 2.2.2. Enzyme Activity Assay in Presence of Compounds **10–12**, **14–19**, **26–28**

**Figure 3:**
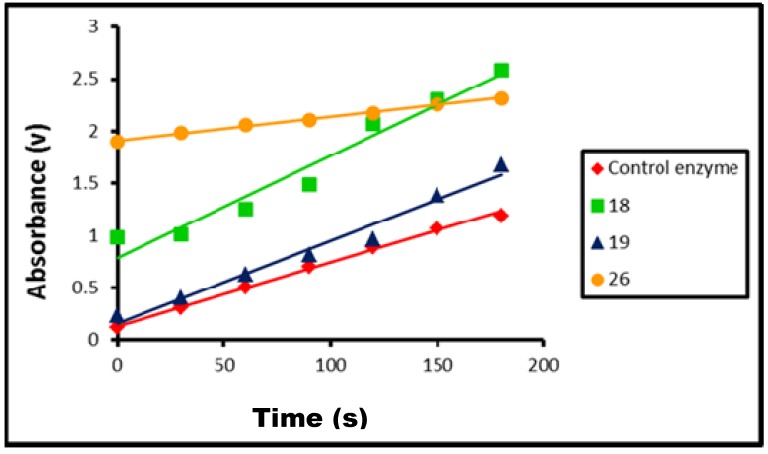
Effect of time on the rate of tyrosinase-catalyzed reaction in presence of compounds **18**, **19**, and **26** compared to control enzyme.

**Figure 4:**
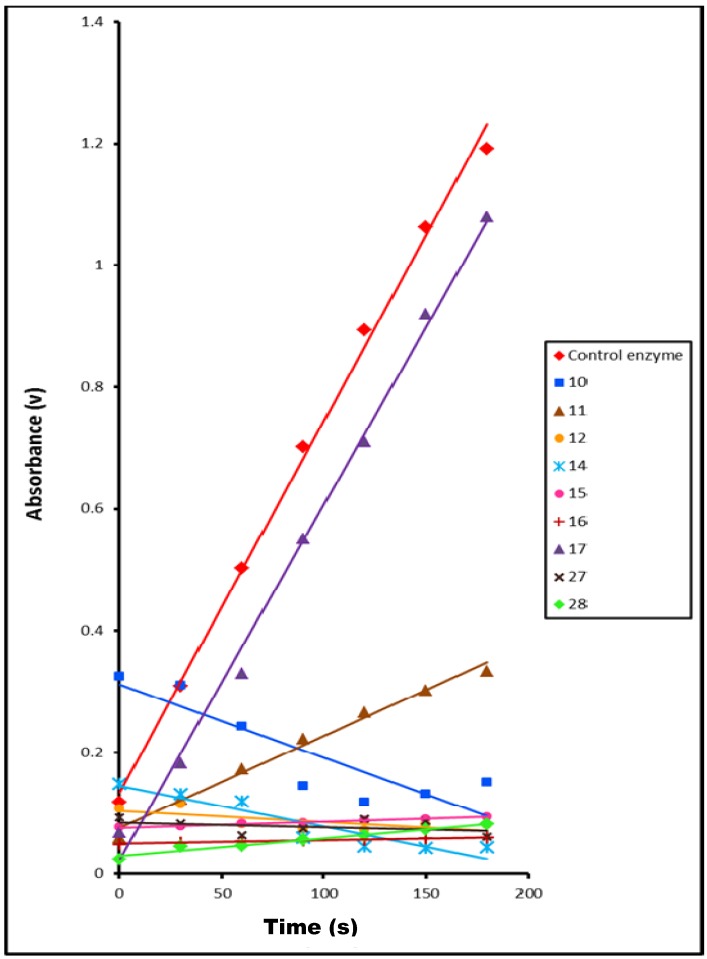
Effect of time on the rate of tyrosinase-catalyzed reaction in presence of compounds **10**–**12**, **14**–**17**, **27** and **28** compared to control enzyme.

The effect of the presence of compounds **10**–**12**, **14**–**19**, **26**–**28** on tyrosinase activity, was determined separately by following the above steps for dopachrome formation then recording the increase in absorbance at 475 nm at time intervals (0–180 s), as shown in Table 2, and Figure 3 and Figure 4. All tests were carried out in duplicate. 

**Table 2:** Effect of time on the velocity of tyrosinase-catalyzed reaction in presence of carbohydrazide derivatives (10–12), (14–19), and (26–28) compared to control enzyme.

Time (s)	Rate (v)												
	Control enzyme	10	11	12	14	15	16	17	18	19	26	27	28
**0**	0.118	0.325	0.0605	0.107	0.148	0.077	0.05	0.069	0.98	0.237	1.897	0.092	0.0245
**30**	0.309	0.31	0.1235	0.115	0.132	0.078	0.052	0.182	1.01	0.402	1.982	0.082	0.0455
**60**	0.502	0.243	0.1725	0.0825	0.119	0.083	0.054	0.329	1.25	0.625	2.056	0.063	0.0465
**90**	0.702	0.145	0.2225	0.073	0.06	0.085	0.055	0.55	1.49	0.818	2.109	0.074	0.0565
**120**	0.893	0.118	0.266	0.0725	0.046	0.088	0.057	0.71	2.07	0.963	2.177	0.089	0.064
**150**	1.063	0.131	0.3015	0.082	0.043	0.091	0.059	0.92	2.3	1.385	2.26	0.086	0.0735
**180**	1.192	0.151	0.3335	0.0805	0.045	0.094	0.06	1.08	2.58	1.68	2.31	0.06	0.082

#### 2.2.3. Results

The obtained results showed that all these compounds are inhibitors for tyrosinase, except for compounds **18**, **19** and **26** which were found to be activators of tyrosinase.

#### 2.2.4. Type of Inhibition

The type of inhibition of *N*'-(1-(4-aminophenyl)ethylidene)-5-formyl-2-methylfuran-3-carbo-hydrazide (**12**), *N*'-(1-(4-aminophenyl)ethylidene)-5-formyl-2-methylfuran-3-carbohydrazide (**16**) and 1-((4-(1-(4-aminophenylethylideneaminocarbamoyl)furan-2-yl)methylene-2-tosylhydrazine (**28**) on enzyme activity was detected by plotting 1/[S] against 1/v using different concentrations of dopa (3, 6, 10, 15, 20 mM) according to the abovementioned steps. Compound **12** showed a highly competitive inhibition, with V_max_ (maximum rate, 0.33) and K_m_ (Michaelis constant, 8.24), while both compound **16** and compound **28** showed an uncompetitive inhibition with V_max_ (0.0667) and K_m_ (0.763), and V_max_ (0.074) and K_m_ (0.444), respectively.

## 3. Experimental

### 3.1. General Methods

Melting points were determined on a Koffler block and are uncorrected. IR spectra were recorded on Perkin Elmer 1600 USA Spectrometer. ^1^H-NMR were recorded on a JEOL JNM ECA 500 MHz instrument using tetramethylsilane as an internal standard. Mass spectra were recorded on a GC-MS solution DI Analysis Shimadzu Qp-2010 instrument. Elemental analysis was determined at the Regional Center for Mycology and Biotechnology, Al-Azhar University Plus. Optical rotation was obtained at 22 °C with a Perkin-Elmer model 241 Polarimeter equipped with a 10 cm, 1 mL micro cell. Thin layer chromatography (TLC) was carried out on silica gel plates. Solutions were evaporated under diminished pressure unless otherwise stated. The ChemDraw-Ultra-8.0 software has been used to name the prepared compounds.

### 3.2. Reactions of Carbohydrazide **2** with Ketones

A solution of 5-(1,2,3,4-tetrahydroxybutyl)-2-methylfuran-3-carbohydrazide **2** (2.5 g, 0.01 mol) [31,32] in ethanol (50 mL) containing AcOH (0.1 mL) was treated with ketone (0.01 mol). The mixture was refluxed for 8 h. After cooling, the product that separated out was filtered off, washed with a little ethanol and dried.

*5-(1*,*2*,*3*,*4-Tetrahydroxybutyl)-2-methyl-N-(1-phenylethylidene)furan-3-carbohydrazide* (**3**). Yield 96.4%. Rwcrystallized from ethanol as canary yellow crystals; m.p. 144–145 °C; R_f_: 0.97 (CHCl_3_/MeOH, 20:1, v/v); [α]_D_^20^ −19.2; IR (KBr): 1564 (C=N), 1642 (CONH), 3055 (NH), 3340 cm^−1^ (OH); ^1^H-NMR (DMSO-*d_6_*) δ: 1.21 (s, 3H, CH_3_CN), 2.34 (s, 3H, CH_3_-furan), 3.33–3.38 (m, 1H, H-3'), 3.41–3.44 (m, 1H, H-2'), 3.47–3.53 (m, 2H, H-4a', H-4b'), 4.28–4.33 (m, 1H, 4'-OH; exchangeable with D_2_O), 4.41 (d, 1H, 3'-OH; *J* = 7.7 Hz, exchangeable with D_2_O), 4.57 (d, 1H, 2'-OH; *J* = 5.4 Hz, exchangeable with D_2_O), 4.69 (d, 1H, H-1'; *J* = 4.6 Hz), 5.06 (d, 1H, 1'-OH; *J* = 5.4 Hz, exchangeable with D_2_O), 7.27 (s, 1H, H-furan), 7.42–7.44 (m, 3H, Ar-H), 7.91–7.93 (m, 2H, Ar-H), 9.87 (s, 1H, NH; exchangeable with D_2_O); Anal. Calcd for C_18_H_22_N_2_O_6_ (362.38): C, 59.66; H, 6.12; N, 7.73 Found: C, 59.50; H, 5.96; N, 7.60.

*5-(1*,*2*,*3*,*4-Tetrahydroxybutyl)-N-(1-(4-hydroxyphenyl)ethylidene)-2-methylfuran-3-carbohydrazide* (**4**). Yield 63.8%. Recrystallized from ethanol as yellow crystals; m.p. 229–230 °C; R_f_: 0.76 (CHCl_3_/MeOH, 20:1, v/v) [α]_D_^20^ −5.5; IR (KBr): 1599 (C=N), 1655 (CONH), 3254, 3322 cm^−1^ (NH and OH); ^1^H-NMR (DMSO-*d_6_*) δ: 2.08 (s, 3H, CH_3_CN), 2.21 (s, 3H, CH_3_-furan), 2.51–2.54 (m, 4H, H-2', H-3', H-4a', H-4b'), 3.42 (bs, 1H, 5'-OH; exchangeable with D_2_O), 4.33–4.34 (m, 1H, 4'-OH; exchangeable with D_2_O), 4.43 (dd, 1H, 3'-OH; *J*_1,2_*=* 7.7 Hz, *J*_1,3_ = 16.8 Hz; exchangeable with D_2_O), 4.57–4.59 (m, 1H, 2'-OH; exchangeable with D_2_O), 4.70 (dd, 1H, H-1'; *J*_1,2_ = 6.1 Hz, *J*_1,3_ = 15.3 Hz), 5.06 (d, 1H, 1'-OH; *J* = 6.9 Hz, exchangeable with D_2_O), 6.69 (s, 1H, H-furan), 6.79 (d, 2H, *o*-OH; *J* = 8.4 Hz), 7.73 (d, 2H, *m*-OH; *J* = 8.4 Hz), 9.73 (bs, 1H, NH; exchangeable with D_2_O); Anal. Calcd for C_18_H_22_N_2_O_7_ (378.38): C, 57.14; H, 5.86; N, 7.40 Found: C, 57.29; H, 6.00; N, 7.56.

*N'-(1-(4-Aminophenyl)ethylidene))-5-(1*,*2*,*3*,*4-tetrahydroxybutyl)-2-methylfuran-3-carbohydrazide* (**5**). Yield 84.9%. Recrystallized from ethanol as golden crystals; m.p. 174–175 °C; R_f_: 0.83 (CHCl_3_/MeOH, 20:1, v/v); [α]_D_^20^ −17; IR (KBr): 1588 (C=N), 1644 (CONH), 3238, 3334, 3387 cm^−1^ (NH, OH, and NH_2_); ^1^H-NMR (DMSO-*d_6_*) δ: 2.19 (s, 3H, CH_3_CN), 2.44 (s, 3H, CH_3_-furan), 3.33–3.38 (m, 1H, H-3'), 3.41–3.44 (m, 1H, H-2'), 3.47–3.49 (m, 1H, H-4a'), 3.50–3.53 (m, 1H, H-4b'), 4.28–4.33 (m, 1H, 4'-OH; exchangeable with D_2_O), 4.41 (d, 1H, 3'-OH; *J* = 7.7 Hz, exchangeable with D_2_O), 4.56 (d, 1H, 2'-OH; *J* = 3.4 Hz, exchangeable with D_2_O), 4.68 (d, 1H, H-1'; *J* = 4.6 Hz), 5.05 (d, 1H, 1'-OH; *J* = 6.8 Hz, exchangeable with D_2_O), 5.44 (s, 2H, NH_2_; exchangeable with D_2_O), 6.54 (d, 2H, *o*-NH_2_), 6.57 (s, 1H, H-furan), 7.58 (d, 2H, *m*-NH_2_), 9 .20 (bs, 1H, NH; exchangeable with D_2_O); MS: *m/z* (%), 77 (4.54), 92 (53.25), 118 (67.81), 133 (41.59),149 (8.57), 210 (9.36), 251 (100), 252 (17.94), 266 (68.55), 267 (13.58, M^+^); Anal. Calcd for C_18_H_23_N_3_O_6_ (377.39): C, 57.29; H, 6.14; N, 11.13 Found: C, 57.40; H, 6.29; N, 11.21.

*5-(1*,*2*,*3*,*4-Tetrahydroxybutyl)-2-methyl–N-(4-methylpentane-2-ylidene)furan-3-carbohydrazide* (**6**). Yield 82%. Recrystallized from ethanol as white crystals; m.p. 142–143 °C; R_f_: 0.55 (CHCl_3_/MeOH, 20:1, v/v); [α]_D_^20^ −8.9; IR (KBr): 1581 (C=N), 1651 (CONH), 3260 (NH), 3321 cm^−1^ (OH); ^1^H-NMR (DMSO-*d_6_*) δ: 0.85 (d, 6H, 2 CH_3_; *J = 6.9 Hz*), 1.83 (s, 3H, CH_3_CN), 1.89–1.90 (m, 1H, CH(CH_3_)_2_), 2.08 (m, 2H, CH_2_), 2.44 (s, 3H, CH_3_-furan), 3.44–3.54 (m, 4H, H-2', H-3', H-4a', H-4b'), 4.34 (d, 1H, 4'-OH; *J* = 5.4 Hz, exchangeable with D_2_O), 4.43 (dd, 1H, 3'-OH; *J*_1,2_ = 7.7 Hz, *J*_1,3_ = 16.8 Hz; exchangeable with D_2_O), 4.58 (t, 1H, 2'-OH; *J*_1,2_ = 7.7 Hz, *J*_1,3_ = 13.8 Hz; exchangeable with D_2_O), 4.70 (dd, 1H, H-1'; *J*_1,2_ = 6.1 Hz, *J*_1,3_ = 15.3 Hz), 5.06 (d, 1H, 1'-OH; *J* = 6.9 Hz, exchangeable with D_2_O), 6.65 (d, 1H, H-furan; *J* = 12.3 Hz), 9.87 (s, 1H, NH; exchangeable with D_2_O); MS: *m/z* (%), 55 (15.90), 57 (46.21), 69 (7.03), 71 (25.45), 95 (6.39), 96 (6.09), 111 (5.61), 113 (14.11), 139 (8.44), 149 (100), 150 (11.54), 167 (30.60), 168 (2.57), 185 (2.07), 230 (2.43), 284 (1.22), 342 (3.66, M^+^). Anal. Calcd for C_16_H_26_N_2_O_6 _(342.39): C, 56.13; H, 7.65; N, 8.18 Found: C, 56.29; H, 7.44; N, 8.30.

### 3.3. Reactions of **3–6** with Acetic Anhydride

5-(1,2,3,4-Tetrahydroxybutyl)-2-methyl-N-(1-arylethylidene)furan-3-carbohydrazides **3**–**5** (0.002 mol) were dissolved in pyridine (10 mL) and acetic anhydride (10 mL) and left for 24 h. The mixture was then poured onto crushed ice, the product that separated was filtered off, washed several times with water and dried.

*5-(1*,*2*,*3*,*4-Tetracetoxybutyl)-2-methyl-N-(1-phenylethylidene)furan-3-carbohydrazide* (**7**). Yield 86%. Recrystallized from ethanol as yellow crystals; m.p. 134–135 °C, R_f_: 0.83 (CHCl_3_/MeOH, 20:1, v/v); [α]_D_^20^ −12.9; IR (KBr): 1594 (C=N), 1654 (CONH), 1720 (CO-acetyl), 3362 cm^−1^ (NH); Anal. Calcd for C_26_H_30_N_2_O_10 _(530.52): C, 58.86; H, 5.70; N, 5.28 Found: C, 58.72; H, 5.52; N, 4.99.

*5-(1*,*2*,*3*,*4-Tetraacetoxybutyl)-N-(1-(4-acetoxyphenyl)ethylidene)-2-methylfuran-3-carbohydrazide* (**8**). Yield 44.9%. Recrystallized from ethanol as pale yellow crystals; m.p. 149–150 °C; R_f_: 0.24 (CHCl_3_/MeOH, 25:1, v/v); [α]_D_^20^ −14.2; IR (KBr): 1579 (C=N), 1635 (CONH), 1754 (CO-acetyl), 3311 cm^−1^ (NH); ^1^H-NMR (CHCl_3_-*d*) δ: 2.17 (s, 3H, CH_3_CO), 2.31 (d, 12H, 4O-Ac), 2.43 (s, 3H, O-Ac), 2.59(s, 3H, CH_3_-furan), 4.44 (m, 2H, H4a', H4b'), 4.45 (dd, 1H, H-3'; *J*_1,2_ = 7.7 Hz, *J*_1,3_ = 16.8 Hz), 4.47 (t, 1H, H-2'; *J*_1,2_ = 7.7 Hz, *J*_1,3_ = 13.8 Hz), 4.58 (dd, 1H, H-1'; *J*_1,2_ = 6.1 Hz, *J*_1,3_ = 15.3 Hz), 6.64 (s, 1H, H-furan), 7.14 (d, 2H, *o*-OAc), 7.93 (d, 2H, *m*-OAc), (s, 1H, NH; exchangeable with D_2_O); Anal. Calcd for C_28_H_32_N_2_O_12 _(588.56): C, 57.14; H, 5.48; N, 4.76 Found: C, 57.28; H, 5.60; N, 4.89.

*N'-(1-(4-Aminophenyl)ethylidene))-5-(1,2,3,4-tetracetoxybutyl)-2-methylfuran-3-carbohydrazide* (**9**). Yield 60.5%, Recrystallized from ethanol as yellow crystals; m.p. 279–280 °C, R_f_: (CHCl_3_/MeOH, 20:1, v/v); [α]_D_^20^ −6.8; IR (KBr): 1602 (C=N), 1660 (CONH), 1732(CO-acetyl), 3288 cm^−1^ (NH); ^1^H-NMR (DMSO-*d_6_*) δ: 2.03 (s, 6H, 2CH_3_), 2.23 (s, 18H, 4O-Ac, N-Ac), 3.34–3.54 (m, 4H, H-1',H-2', H-3', H4a,4b'), 6.64 (s, 1H, H-furan), 7.62 (d, 2H, Ar-H; *J* = 8.4 Hz), 7.82 (d, 2H, Ar-H; *J* = 8.4 Hz), 9.03 (s, 1H, NH; exchangeable with D_2_O), 10.08 (s, 1H, NH; exchangeable with D_2_O). Anal. Calcd for C_28_H_33_N_3_O_11 _(587.58): C, 57.24; H, 5.66; N, 7.15 Found: C, 57.02; H, 5.49; N, 7.02.

### 3.4. Periodate Oxidation of **3–6**

A solution of **3**–**6** (0.003 mol) dissolved in distilled water (50 mL) was treated with a solution of NaIO_4_ (0.008 mol) in distilled water (50 mL) dropwise with stirring for 3 h, the product that separated out was filtered off, washed with water and dried.

*5-Formyl-2-methyl-N'-(1-phenylethylidene) furan-3-carbohydrazide * (**10**). Yield 65%. Recrystallized from EtOH as a yellow powder; m.p. 114–115 °C; R_f_: 0.84 (CHCl_3_/MeOH, 20:1, v/v); IR (KBr): 1593 (C=N), 1643 (CONH), 1728 (CHO), 3236 cm^−1^ (NH); ^1^H-NMR (DMSO-*d_6_*) δ: 2.23 (s, 6H, 2CH_3_), 7.42–7.43 (m, 2H, Ar-H), 7.31 (s, 1H, H-furan), 7.87–7.93 (m, 3H, Ar-H), 9.23 (bs, 1H, NH; exchangeable with D_2_O), 9.87 (s, 1H, CHO); Anal. Calcd for C_15_H_14_N_2_O_3_ (270.28): C, 66.66; H, 5.22; N, 10.36 Found: C, 66.44; H, 4.99; N, 10.19.

*N'-(1-(4-Hydroxyphenyl)ethylidene)-5-formyl-2-methylfuran-3-carbohydrazide* (**11**). Yield 43.5%. Recrystallized from EtOH as white crystals; m.p. 192–193 °C; R_f_: 0.32 (CHCl_3_/MeOH, 25:1, v/v); IR (KBr): 1597 (C=N), 1668 (CONH), 1751 (CHO), 3251, 3404 cm^−1^ (NH and OH); ^1^H-NMR (DMSO-*d_6_*) δ: 2.27 (s, 6H, 2 CH_3_), 4.91 (bs,1H, OH; exchangeable with D_2_O), 6.86 (d, 2H, *o*-OH; *J* = 8.4 Hz), 7.81(d, 2H, *m*-OH; *J* = 8.4 Hz), 8.91 (s, 1H, NH; exchangeable with D_2_O), 9.34 (s, 1H, CHO); Anal. Calcd for C_15_H_14_N_2_O_4_ (286.28): C, 62.93; H, 4.93; N, 9.79 Found: C, 62.75; H, 4.77; N, 9.60.

*N'-(1-(4-Aminophenyl)ethylidene)-5-formyl-2-methylfuran-3-carbohydrazide* (**12**). Yield 36%. Rerystallized from EtOH as dark yellow crystals; m.p. 145–146 °C; R_f_: 0.86 (CHCl_3_/MeOH, 20:1, v/v); IR (KBr): 1586 (C=N), 1652 (CONH), 1768 (CHO), 3233, 3322, 3344 cm^−1^ (NH and NH_2_); ^1^H-NMR (DMSO-*d_6_*) δ: 2.27 (s, 6H, 2CH_3_), 4.95 (bs, 2H, NH_2_); exchangeable with D_2_O), 6.65–6.70 (m, 2H, *o*-NH_2_), 6.58 (s, 1H, H-furan), 7.69–7.73 (m, 2H, *m*-NH_2_), 9.36 (s, 1H, NH; exchangeable with D_2_O), 9.44 (s, 1H, CHO); Anal. Calcd for C_15_H_15_N_3_O_3_ (285.3): C, 63.15; H, 5.30; N, 14.73 Found: C, 62.99; H, 5.22; N, 14.62.

*5-Formyl-2-methyl-N'-(4-methylpentan-2-ylidene)furan-3-carbohydrazide* (**13**). Yield 58%. Recrystallized from EtOH as pale yellow needles; m.p. 210–211 °C; R_f_: 0.77 (*n*-hexane/EtOAc, 7:1, v/v); IR (KBr): 1632 (C=N), 1666 (CONH), 1720 (CHO), 3437 cm^−1^ (NH); MS: *m/z* (%), 65 (48.47), 80 (33.29), 92 (40.53), 93 (6.69), 104 (7.80), 113 (5.29), 117 (11), 118 (72.01), 119 (33.98), 122 (8.50), 132 (11.42), 133 (51.67), 136 (4.46), 141 (16.43), 145 (14.62), 148 (32.17), 149 (80.22), 150 (5.99), 158 (16.85), 167 (23.68), 174 (12.26), 178 (17.27), 181 (39.42), 182 (23.54), 193 (16.16), 195 (17.97), 196 (15.46), 211 (17.97), 224 (18.11), 225 (15.32), 227 (15.46), 230 (15.88), 250 (100, M^+^); Anal. Calcd for C_13_H_18_N_2_O_3_ (250.29): C, 62.38; H, 7.25; N, 11.19 Found: C, 62.13; H, 7.02; N, 11.10.

### 3.5. Reactions of 5-Formyl-2-methyl-N'-(1-arylethylidene) furan-3-carbohydrazide **10–12** with Thio-semicarbazide Derivatives

A solution of 5-formyl-2-methyl-N'-(1-arylethylidene)furan-3-carbohydrazide **10**–**12** (0.001 mol) in ethanol (20 mL) containing acetic acid (0.01 mL) was treated with thiosemicarbazide or *p*-tolyl- or *o*-tolylthiosemicarbazide (0.001 mol). The mixture was refluxed for 3–6 h. After cooling, the thiosemicarbazone which separated out was filtered off, washed with little ethanol and dried.

*1-((4-(1-Phenylethylideneaminocarbamoyl)-5-methylfuran-2-yl)methylene)thiosemicarbazide* (**14**). Yield 98%. Recrystallized from ethanol as yellow needles; m.p. 159–160 °C; R_f_: 0.83(CHCl_3_/MeOH, 20:1, v/v); IR (KBr): 1489 (CSNH), 1589 (C=N), 1684 (CONH), 3148, 3207 (2NH), 3362, 3405 cm^−1^ (NH_2_); ^1^H-NMR (CHCl_3_-*d*) δ: 1.83 (bs, 2H, NH_2_; exchangeable with D_2_O), 2.29 (s, 3H, CH_3_CN), 2.31 (s, 3H, CH_3_-furan), 6.51 (bs, 1H, NH; exchangeable with D_2_O), 7.35–7.47 (m, 5H, Ar-H), 7.69 (s, 1H, H-furan), 7.91 (s, 1H, CH=N), 8.79 (s, 1H, NH; exchangeable with D_2_O); MS: *m/z* (%), 51 (21.35), 76 (6.26), 77 (82.55), 91 (9.79), 92 (4.78), 103 (22.11), 118 (32.79), 133 (11.33), 221 (100), 222 (21.64), 343 (19.01, M^+^); Anal. Calcd for C_16_H_17_N_5_O_2_S (343.4): C, 55.96; H, 4.99; N, 20.39 Found: C, 55.79; H, 4.84; N, 20.22.

*1-((4-(1-(4-Hydroxyphenylethylideneaminocarbamoyl)furan-2-yl)methylene)thiosemi-carbazide* (**15**). Yield 99.0%. Recrystallized from ethanol as yellow crystals; m.p. 146–147 °C; R_f_: 0.47 (CHCl_3_/MeOH, 25:1, v/v); IR (KBr): 1499 (CSNH), 1585 (C=N), 1654 (CONH), 3182, 3200, 3358 cm^−1^ (2NH, NH_2_, and OH); ^1^H-NMR (DMSO-*d_6_*) δ: 2.19 (s, 6H, 2CH_3_), 4.34 (bs, 2H, NH_2_; exchangeable with D_2_O), 6.72 (d, 2H, *o*-OH; *J* = 8.4 Hz), 6.76 (d, 1H, H-furan; *J* = 6.9 Hz), 7.73 (d, 2H, *m*-OH; *J* = 8.4 Hz), 7.77 (s, 1H, CH=N), 8.12 (s, 1H, OH; exchangeable with D_2_O), 9.73 (bs, 1H, NH; exchangeable with D_2_O), 10.02 (s, 1H, NH; exchangeable with D_2_O); Anal. Calcd for C_16_H_17_N_5_O_3_S (359.4): C, 53.47; H, 4.77; N, 19.49 Found: C, 53.36; H, 4.59; N, 19.34.

*1-((4-(1-(4-Aminophenylethylideneaminocarbamoyl)furan-2-yl)methylene)thiosemicarbazide* (**16**). Yield 43%. Recrystallized from ethanol as orange crystals; m.p. 179–180 °C; R_f_: 0.75 (CHCl_3_/MeOH, 20:1, v/v); IR (KBr): 1489 (CSNH), 1591 (C=N), 1652 (CONH), 3311, 3388, 3344 cm^−1^ (NH, NH_2_); ^1^H-NMR [(CH_3_)_2_CO-*d_6_*] δ: 2.28 (s, 6H, 2CH_3_), 4.99 (bs, 4H, 2NH_2_; exchangeable with D_2_O), 6.61 (d, 2H, *o*-NH_2_; *J* = 8.4 Hz), 6.63 (s, 1H, H-furan), 7.40 (bs,1H, NH; exchangeable with D_2_O), 7.61 (d, 2H, *m*-NH_2_; *J* = 8.4 Hz), 7.70 (s,1H, CH=N), 9.23 (s,1H, NH; exchangeable with D_2_O); MS: *m/z* (%),64 (7.85), 65 (62.80), 77 (5.46), 80 (5.78), 91(26.67), 92 (55.68), 106 (7.60), 107 (7.39), 118 (71.91), 119 (38.57), 133 (61.38), 134 (17.57), 148 (15.14), 174 (4.71), 191 (27.20), 208 (30.73), 209 (4.70), 210 (9.01), 251 (100), 252 (18.49), 266 (66.40),358 (17.57, M^+^); Anal. Calcd for C_16_H_18_N_6_O_2_S (358.42): C, 53.62; H, 5.06; N, 23.45 Found: C, 53.41; H, 4.97; N, 23.22.

*1-((4-(1-Phenylethylideneaminocarbamoyl)-5-methylfuran-2-yl)methylene)-4-o-tolylthiosemicarbazide* (**17**). Yield 68.8%/ Recrystallized from ethanol as white needles; m.p. 157–158 °C; R_f_: 0.91 (CHCl_3_/MeOH, 20:1, v/v); IR (KBr): 1488 (CSNH), 1602 (C=N), 1658 (CONH), 3220, 3293 cm^−1^ (2NH); ^1^H-NMR (CHCl_3_-*d*) δ: 2.35 (s, 9H, 3CH_3_),7.21 (s, 1H, H-furan), 7.23 (s, 1H, CH=N), 7.25–7.27 (m, 2H, Ar-H), 7.41–7.46 (m, 4H, Ar-H), 7.73–7.75 (m, 3H, Ar-H), 8.96 (s, 1H, NH; exchangeable with D_2_O), 9.21 (s, 2H, 2NH; exchangeable with D_2_O); MS: *m/z* (%), 65 (28.65), 77 (100), 91 (32.72), 103 (16.00), 106 (25.49), 107 (51.97), 118 (24.99), 133 (64.04), 134 (19.96), 150 (15.79), 151 (9.96), 164 (11.37), 165 (8.88), 268 (61.82), 283 (52.96), 284 (10.21), 433 (8.86, M^+^);Anal. Calcd for C_23_H_23_N_5_O_2_S (433.53): C, 63.72; H, 5.35; N, 16.15 Found: C, 63.47; H, 5.11; N, 15.90.

*1-((4-(1-(4-Hydroxyphenylethylideneaminocarbamoyl)-5-methylfuran-2-yl)methylene)-4-o-tolylthio-semicarbazide* (**18**). Yield 53%. Recrystallized from ethanol as yellow crystals; m.p. 229–230 °C; R_f_: 0.5 (CHCl_3_/MeOH, 25:1, V/V); IR (KBr): 1486 (CSNH), 1613 (C=N), 1664 (CONH), 3235, 3323, 3462 cm^−1^ (2NH, OH);^ 1^H-NMR (DMSO-*d_6_*) δ: 2.19 (s, 3H, CH_3_CN), 2.21 (s, 3H, CH_3_-fursn), 2.28 (s, 3H, CH_3_–tolyl), 6.74 (s,1H, H-furan), 5.68 (s, 1H, OH; exchangeable with D_2_O), 6.79 (d, 2H, *o*-OH; *J* = 8.4 Hz), 7.13–7.20 (m, 2H, *o*-tolyl), 7.23 (d, 1H, *o*-tolyl; *J* = 6.9 Hz), 7.32 (d, 1H, *o*-tolyl; *J* = 7.7 Hz), 7.73 (d, 2H, *m*-OH; *J* = 8.4 Hz), 7.84 (s, 1H, CH=N), 9.78 (bs, 3H, 3NH; exchangeable with D_2_O); MS: *m/z* (%), 50 (10.85), 51 (24.80), 65 (90.46), 77 (74.51), 91 (70.32), 107 (100), 119 (51.20), 134 (57.54), 149 (28.11), 150 (15.98), 164 (10.61), 175 (10.30), 205 (6.06), 212 (8.73), 237 (6.29), 253 (97.99), 268 (80.70), 283 (26.17), 284 (9.88), 296 (6.67), 299 (6.04), 449 (10.61, M^+^); Anal. Calcd for C_23_H_23_N_5_O_3_S (449.53): C, 61.45; H, 5.16; N, 15.58 Found: C, 61.23; H, 5.02; N, 15.40.

*1-((4-(1-(4-Aminophenylethylideneaminocarbamoyl)-5-methylfuran-2-yl)methylene)-4-o-tolylthio-semicarbazide* (**19**). Yield 44%. Recrystallized from ethanol as yellow crystals; m.p. 139–140 °C; R_f_: 0.77 (CHCl_3_/MeOH, 20:1, v/v); IR (KBr): 1482 (CSNH), 1622 (C=N), 1683 (CONH), 3205, 3252, 3288, 3324 cm^−1^ (3NH, NH_2_); ^1^H-NMR [(CH_3_)_2_CO-*d_6_*] δ: 2.20 (s, 3H, CH_3_CN), 2.24 (s, 3H, CH_3_-furan), 2.35 (s, 3H, CH_3_–tolyl), 5.45 (bs, 2H, NH_2_; exchangeable with D_2_O), 6.52 (d, 2H, *o*-NH_2_; *J* = 8.4 Hz), 6.56 (s, 1H, H-furan), 7.16–7.19 (m, 2H, *o*-tolyl), 7.23 (d, 1H, *o*-tolyl; *J* = 6.9 Hz), 7.37 (d, 1H, *o*-tolyl; *J* = 7.7 Hz), 7.6 (s, 1H, CH=N), 7.69 (d, 2H, *m*-NH_2_; *J* = 8.4 Hz), 9.71 (s, 1H, NH; exchangeable with D_2_O), 10.27 (s, 1H, NH; exchangeable with D_2_O); Anal. Calcd for C_23_H_24_N_6_O_2_S (448.54): C, 61.59; H, 5.39; N, 18.74 Found: C, 61.44; H, 5.28; N, 18.66.

### 3.6. Reactions of Thiosemicarbazones **14–19** with Acetic Anhydride

A mixture of **14**–**19** (0.01 mol), acetic anhydride (10 mL, 0.1 mol) was gently refluxed for 2 h. The hot solution was poured onto ice water (10 mL) and the dihydro-1,3,4-thiadiazole which separated was filtered off, washed several times by water and dried.

*5-(5-Acetamido-4-acetyl-4,5-dihydro-1,3,4-thiadiazol-2-yl)-2-methyl-N'-(1-phenylethylidene)furan-3-carbohydrazide* (**20**). Yield 65%. Recrystallized from ethanol as white needles; m.p. 220–221 °C; R_f_: 0.65 (CHCl_3_/MeOH, 25:1, v/v); IR (KBr): 1597 (C=N), 1698 (CONH), 1715 (CO-acetyl), 3135, 3219 cm^−1^ (2NH); ^1^H-NMR (DMSO-*d_6_*) δ: 1.98 (s, 3H, CH_3_C=N), 2.16 (s, 6H, 2 N-Ac), 2.25(s, 3H, CH_3_-furan), 7.21 (d, 1H, H-furan), 7.23 (s, 1H, H-thiadiazolyl), 7.30–7.32 (m, 5H, Ar-H), 9.23 (bs, 1H, NH; exchangeable with D_2_O), 11.60 (s, 1H, NH; exchangeable with D_2_O); MS: *m/z* (%), 59 (6.70), 77 (29.21), 78 (9.60), 91 (5.22), 92 (5.37), 103 (14.25), 104 (13.30), 116 (9.86), 117 (6.74), 118 (25.31), 119 (7.62), 120 (4.38), 121 (12.26), 133 (15.23), 134 (5.36), 150 (5.09), 158 (12.76), 178 (32.05), 220 (100), 221 (13.32), 222 (5.74), 235 (13.82), 262 (13.77), 277 (28.09), 427 (5.09, M^+^); Anal. Calcd for C_20_H_21_N_5_O_4_S (427.48): C, 56.19; H, 4.95; N, 16.38 Found: C, 55.96; H, 4.81; N, 16.14.

*4-(1-(5-(5-(N-Acetylacetamido)-4-acetyl-4,5-dihydro-1,3,4-thiadiazol-2-yl)-2-methylfuran-3-carboyl imino)ethyl)phenylacetate* (**21**). Yield 42%. Recrystallized from ethanol as white crystals; m.p. 123–124 °C; R_f_: 0.41 (*n*-hexane/EtOAc, 7:1, v/v); IR (KBr): 1593 (C=N), 1671 (CONH), 1680 (CO-acetyl), 3237 cm^−1^ (NH); ^1^H-NMR (DMSO-*d_6_*) δ: 1.24 (s, 3H, CH_3_C=N), 2.14 (s, 3H, N-Ac), 2.21 (s, 3H, O-Ac), 2.27 (s, 3H, CH_3_-furan), 2.34 (s, 6H, N-(Ac)_2_), 6.69 (s, 1H, H-furan), 7.06 (s, 1H, H-thiadiazolyl), 7.23–7.26 (m, 4H, Ar-H), 8.22 (s, 1H, NH; exchangeable with D_2_O); Anal. Calcd for C_24_H_25_N_5_O_7_S (527.55): C, 54.64; H, 4.78; N, 13.28 Found: C, 54.52; H, 4.64; N, 13.17.

*5-(5-(N-Acetylacetamido)-4-acetyl-4,5-dihydro-1,3,4-thiadiazol-2-yl)-N'-(1-(4-acetamidophenyl)-ethylidene)-2-methylfuran-3-carbohydrazide* (**22**). Yield 73%. Recrystallized from ethanol as buff needles; m.p. 152–135 °C; R_f_: 0.47 (*n*-hexane/EtOAc, 7:1, v/v); IR (KBr): 1610 (C=N), 1651 (CONH), 1695 (CO-acetyl), 3232, 3345 cm^−1^ (2NH); ^1^H-NMR [(CH_3_)_2_CO-*d_6_*] δ: 2.03 (s, 3H, CH_3_CN), 2.08 (s, 3H, N-Ac), 2.11 (s, 3H, N-Ac), 2.29 (s, 3H, CH_3_-furan), 2.48(s, 6H, N(Ac)_2_), 7.22 (d, 1H, H-furan), 7.30 (d, 2H, *m*-NAc; *J* = 8.4 Hz), 7.44 (s, 1H, H-thiadiazolyl), 7.56 (d, 2H, *o*-NAc; *J* = 8.4 Hz), 9.23 (s, 1H, NH; exchangeable with D_2_O), 10.50 (bs, 1H, NH-Ac; exchangeable with D_2_O); MS: *m/z* (%), 56 (31.73), 57 (20.17), 59 (30,76), 60 (21.91), 63 (7.39), 67 (12.24), 74 (60.64), 80 (5.05) 81 (8.21), 104 (7.24), 105 (5.83), 111 (7.53), 114 (16.47), 115 (100), 134 (13.51), 135 (12.88), 144 (6.56), 146 (4.86), 157 (15.11), 168 (5.88), 172 (5.88), 182 (5.73), 203 (6.66), 215 (7.19), 222 (5.00), 223 (5.49), 251 (9.33), 255 (8.41), 257 (4.71), 277 (17.15), 282 (5.78), 286 (5.34), 295 (5.49), 319 (5.78), 526 (5.34, M^+^); Anal. Calcd for C_24_H_26_N_6_O_6_S (526.56): C, 54.74; H, 4.98; N, 15.96 Found: C, 54.49; H, 4.81; N, 15.70.

*5-(5-(N-o-Tolylacetamido)-4-acetyl-4,5-dihydro-1,3,4-thiadiazol-2-yl)-N-acetyl-2-methyl-N'-(1-phenyl ethylidene)furan-3-carbohydrazide* (**23**). Yield 59%. Recrystallized from ethanol as white needles; m.p. 107–108 °C; R_f_: 0.87 (CHCl_3_/MeOH, 25:1, v/v); IR (KBr): 1594 (C=N), 1682 cm^−1^ (CO-acetyl); ^1^H-NMR (CHCl_3_-*d*)δ: 1.88 (s, 6H, 2N-Ac), 2.20 (s, 3H, CH_3_CN), 2.28 (s, 3H, CH_3_–furan), 2.35 (s, 3H, CH_3_*-o*-tolyl), 2.38 (s, 3H, N-Ac), 6.81 (s, 1H, H- furan), 7.17 (s, 1H, H-thiadiazolyl), 7.21–7.25 (m, 3H, Ar-H), 7.31–7.40 (m, 6H, Ar-H); MS: *m/z* (%),65 (7.53), 77 (27.45), 78 (9.15), 91 (17.54), 103 (13.66), 104 (18.10), 107 (23.20), 118 (28.32), 121 (19.42), 133 (18.36), 149 (7.33), 150 (7.61), 161 (13.18), 206 (11.26), 221 (0.67), 248 (14.33), 250 (9.44), 268 (45.35), 310 (100), 311 (20.21), 325 (17.89), 367 (47.84), 559(13.18, M^+^); Anal. Calcd for C_29_H_29_N_5_O_5_S (559.64): C, 62.24; H, 5.22; N, 12.51 Found: C, 62.50; H, 4.99; N, 12.38.

*4-(1-(5-(5-(N-o-Tolylacetamido)-4-acetyl-4,5-dihydro-1,3,4-thiadiazol-2-yl)-2-methylfuran-3-carboyl-imino)ethyl)phenyl acetate* (**24**). Yield 40%. Recrystallized from ethanol as buff needles; m.p. 119–120 °C; R_f_: 0.28 (CHCl_3_/MeOH, 25:1, v/v); IR (KBr): 1560 (C=N), 1652 (CONH), 1699 (CO-acetyl), 3233 cm^−1^ (NH); ^1^H-NMR (DMSO-*d_6_*) δ: 1.71 (s, 3H, CH_3_CN), 1.79 (s, 6H, N-Ac, O-Ac), 1.87 (s, 3H, CH_3_–furan), 1.93 (s, 3H, CH_3_–*o*-tolyl), 6.72 (s, 1H, H-furan), 6.78 (d, 2H, *o*-OAc; *J* = 8.4 Hz), 7.13–7.20 (m, 2H, *o*-tolyl-H), 7.21 (d, 1H, *o*-tolyl-H; *J* = 6.9 Hz ),7.29 (d, 1H, *o*-tolyl-H; *J* = 7.7 Hz), 7.70 (d, 2H, *m*-OAc; *J* = 8.4 Hz), 7.81 (s, 1H, H-thiadiazolyl), 8.85 (s, 1H, NH; exchangeable with D_2_O), 9.69 (s, 1H, NH; exchangeable with D_2_O). Anal. Calcd for C_29_H_29_N_5_O_6_S (533.6): C, 60.51; H, 5.08; N, 12.17 Found: C, 60.37; H, 4.99; N, 12.05.

*5-(5-(N-o-Tolylacetamido)-4-acetyl-4,5-dihydro-1,3,4-thiadiazol-2-yl)-N'-(1-(4-(N-acetylacetamido) phenyl)ethylidene)-2-methylfuran-3-carbohydrazide* (**25**). Yield 69%. Recrystallized from ethanol as yellow needles; m.p. 114–115 °C; R_f_: 0.65 (CHCl_3_/MeOH, 25:1, v/v); IR (KBr): 1598 (C=N), 1647 (CONH), 1677 (CO-acetyl), 3377 cm^−1^ (NH); Anal. Calcd for C_31_H_32_N_6_O_6_S (616.69): C, 60.38; H, 5.23; N, 13.63 Found: C, 60.26; H, 5.31; N, 13.74.

### 3.7. Reactions of 5-Formyl-2-methyl-N'-(1-arylethylidene)furan-3-carbohydrazides **10–12** with p-tosylhydrazine

A solution of 5-formyl-2-methyl-N'-(1-arylethylidene)furan-3-carbohydrazide **10**–**12** (0.001 mol) in ethanol (30 mL) containing acetic acid (0.01 mL) was treated with *p*-tosylhydrazine (0.196 g, 0.001 mol). The mixture was refluxed for 3–4 h. After cooling, the product which separated out was filtered off, washed with little ethanol and dried.

*1-((4-(1-Phenylethylideneaminocarbamoyl)furan-2-yl)-2-p-tosylhydrazine methylene* (**26**). Yield 57%. Recrystallized from ethanol as white crystals; m.p. 115–116 °C; R_f_: 0.88 (CHCl_3_/MeOH, 20:1, v/v); IR (KBr): 1163,1335 (SO_2_), 1599 (C=N), 1659 (CONH), 3223 cm^−1^ (NH); ^1^H-NMR [(CH_3_)_2_CO-*d_6_*] δ: 2.21 (s, 3H, CH_3_CN), 2.30 (s, 3H, CH_3_-furan), 2.36 (s, 3H, CH_3_–tolyl), 7.32–7.36 (m, 3H, Ph), 7.41–7.43 (m, 2H, *m*-H of Ph), 7.68–7.69 (m, 2H,*o*-H of *p*-tolyl), 7.83 (s,1H, H-furan), 7.85 (s, 1H, CH=N), 7.93–7.95 (m, 2H, *m*-H of *p*-tolyl), 9.36 (s, 2H, 2NH; exchangeable with D_2_O); MS: *m/z* (%), 65 (40.65), 77 (30.59), 78 (25.23), 91 (33.65), 92 (90.68), 104 (100), 118 (20.56), 132 (30.51), 133 (85.78), 134 (10.56), 140 (5.36), 288 (8.96), 438 (10.56, M^+^). Anal. Calcd for C_22_H_22_N_4_O_4_S (438.5): C, 60.26; H, 5.06; N, 12.78 Found: C, 60.05; H, 4.93; N, 12.53.

*1-((4-(1-(4-Hydroxyphenylethylideneaminocarbamoyl)furan-2-yl)methylene-2-p-tosylhydrazine * (**27**). Yield 42%. Recrystallized from ethanol as orange crystals; m.p. 269–270 °C, R_f_: 0.92 (CHCl_3_/MeOH, 15:1, v/v); IR (KBr): 1164, 1337 (SO_2_), 1598 (C=N), 1658 (CONH), 3225, 3447 cm^−1^ (NH, OH); ^1^H-NMR (DMSO-*d_6_*) δ: 2.14 (s, 3H, CH_3_CN), 2.32 (s, 6H, CH_3_-furan, CH_3_ of *p*-tolyl), 4.99 (bs, 1H, OH; exchangeable with D_2_O), 6.70 (d,1H, H-furan; *J* = 8.5 Hz), 7.37 (d, 2H,*o*-H of *p*-tolyl; *J* = 7.7 Hz), 7.33 (t, 2H, *o*-OH; *J*_1,2_ = 3.1, *J*_1,3_ = 5.4 Hz), 7.58 (t, 2H, *m*-OH; *J*_1,2_ = 3.1, *J*_1,3_ = 5.4 Hz), 7.69 (s, 1H, CH=N), 7.78 (d, 2H,*m*-H of *p*-tolyl;*J* = 7.7 Hz), 9.71 (s, H, NH; exchangeable with D_2_O),10.47 (s, H, NH; exchangeable with D_2_O); MS: *m/z* (%), 59 (7.33), 81 (4.23), 108 (19.85), 119 (11,00), 120 (21.47), 121 (16.05), 149 (31.06), 150 (7.52), 155 (6.25), 156 (6.25), 158 (12.69), 178 (27.39), 220 (100), 221 (13.15), 235 (13.28), 262 (15.08), 277 (29.17), 278 (6.49), 454 (15.08, M^+^). Anal. Calcd for C_22_H_22_N_4_O_5_S (454.5): C, 58.14; H, 4.88; N, 12.33 Found: C, 57.96; H, 4.75; N, 12.22.

*1-((4-(1-(4-Aminophenylethylideneaminocarbamoyl)furan-2-yl)methylene-2-p-tosylhydrazine * (**28**). Yield 83%. Recrystallized from ethanol as yellow crystals, m.p. 160–162 °C; R_f_: 0.61 (CHCl_3_/MeOH, 25:1, v/v); IR (KBr): 1182, 1363 (SO_2_), 1589 (C=N), 1661 (CONH), 3238, 3391, 3429 cm^−1^ (NH, NH_2_). Anal. Calcd for C_22_H_23_N_5_O_4_S (453.51): C, 58.26; H, 5.11; N, 15.44 Found: C, 58.19; H, 5.02; N, 15.40.

### 3.8. Reactions of **26–28** with Acetic Anhydride

A mixture of 1-((4-(1-arylethylidene aminocarbamoyl)furan-2-yl)methylene-2-*p*-tosylhydrazine **26**–**28** (0.0005 mol), acetic anhydride (20 mL) was gently refluxed for 20 min. The hot solution was poured onto ice water (10 mL), the 1,2,3,4-oxathiadiazole product which separated was filtered off, washed several times by water and dried.

*2-Methyl-N'-(1-phenylethylidene)-5-(2-p-tolyl-1*,*2*,*3*,*4-oxathiadiazol-5-yl)furan-3-carbohydrazide *(**29**). Yield 54%. Recrystallized from ethanol as buff needles; m.p. 174–175 °C; R_f_: 0.83 (CHCl_3_/MeOH, 25:1, v/v); IR (KBr): 1598 (C=N), 1645 (CONH), 1710 (CO-acetyl), 3260 cm^−1^ (NH); ^1^H-NMR (CHCl_3_-*d*) δ: 2.05 (s, 3H, CH_3_CN), 2.40 (s, 3H, CH_3_–furan), 2.43 (s, 3H, CH_3_*p*-tolyl), 7.26–7.33 (m, 4H, *p*-tolyl), 7.40 (s,1H, H-furan), 7.44–7.48 (m, 1H, *p*-H of Ph), 7.76 (d, 2H, *m*-H of Ph;*J* = 8.4 Hz), 7.94 (d, 2H, *o*-H of Ph; *J *= 8.4 Hz), 8.19 (bs, 1H, NH; exchangeable with D_2_O); MS: *m/z* (%), 51 (15.47), 65 (24.48), 77 (47.41), 91 (55.26), 103 (96.40), 104 (22.97), 105 (21.46), 119 (12.93), 133 (100), 134 (9.78), 139 (17.48), 160 (78.93), 175 (55.76), 420 (9.77, M^+^); Anal. Calcd for C_22_H_20_N_4_O_3_S (420.48): C, 62.84; H, 4.79; N, 13.32 Found: C, 62.95; H, 4.59; N, 13.19.

*4-(1-(2-Methyl-5-(2-p-tolyl-1*,*2*,*3*,*4-oxathiadiazol-5-yl)furan-3-carboylimino)ethyl) phenyl acetate* (**30**). Yield 42%. Recrystallized from ethanol as yellow needles; m.p. 104–105 °C; R_f_: 0.26 (CHCl_3_/MeOH, 25:1, v/v); IR (KBr): 1597 (C=N), 1643 (CONH), 1713 (CO-acetyl), 3368 cm^-1^ (NH); MS: *m/z* (%), 51 (15.47), 65 (91.77), 77 (78.93), 91 (71.33), 103 (9.40), 105 (23.11), 107 (100), 119 (63.07), 133 (60.24), 134 (65.21), 149 (36.02), 150 (23.07), 160 (9.03), 164 (45.44), 175 (12.24), 253 (96.40), 261 (0.21), 267 (11.78), 268 (45.26), 283 (23.05), 296 (12.45, 478 (24.48, M^+^); Anal. Calcd for C_24_H_22_N_4_O_5_S (478.52): C, 60.24; H, 4.63; N, 11.71 Found: C, 60.07; H, 4.54; N, 11.60.

*N'-(1-(4-Acetamidophenyl)ethylidene)-2-methyl-5-(2-p-tolyl-1*,*2*,*3*,*4-oxathiadiazol-5-yl)furan-3-carbo- hydrazide *(**31**). Yield 32%. Recrystallized from ethanol as yellow needles; m.p. 111–112 °C; R_f_: 0.58 (CHCl_3_/MeOH, 25:1, v/v); IR (KBr): 1599 (C=N), 1645 (CONH), 1710 (CO-acetyl), 3390 (NH); ^1^H-NMR [(CH_3_)_2_CO-*d_6_*] δ: 2.09 (s, 6H, CH_3_CN, CH_3_CO), 2.49 (s, 3H, CH_3_–furan), 2.49 (s, 3H, CH_3_*p*-tolyl), 7.64 (s, 1H, H-furan), 7.73 (d, 4H, *p*-tolyl; *J* = 9.2 Hz), 7.90 (d, 4H, Ar-H; *J* = 8.4 Hz), 9.57 (bs, 2H, 2NH; exchangeable with D_2_O); Anal. Calcd for C_24_H_23_N_5_O_4_S (477.54): C, 60.36; H, 4.85; N, 14.67 Found: C, 60.19; H, 4.71; N, 14.63.

## 4. Conclusions

Some new *C*-nucleoside derivatives, thiadiazole and oxathiadiazole derivatives have been prepared as well as their physical properties and biological effect on the enzyme tyrosinase studied. 
